# Eco-friendly synthesis of silver nanoparticles from peel and juice *C. limon* and their antiviral efficacy against HSV-1 and SARS-CoV-2

**DOI:** 10.1016/j.virusres.2024.199455

**Published:** 2024-08-24

**Authors:** Federica Dell'Annunziata, Ekaterine Mosidze, Veronica Folliero, Erwin P. Lamparelli, Valentina Lopardo, Pasquale Pagliano, Giovanna Della Porta, Massimiliano Galdiero, Aliosha Dzh Bakuridze, Gianluigi Franci

**Affiliations:** a Department of Medicine, Surgery and Dentistry "Scuola Medica Salernitana", University of Salerno, 84081 Baronissi, Italy; b Department of Experimental Medicine, University of Campania "Luigi Vanvitelli", 80138 Naples, Italy; c Department of Pharmaceutical Technology, Tbilisi State Medical University, 33 Vazha-Pshavela Ave, Tbilisi, 0178, Georgia; d UOC Patologia e Microbiologia, San Giovanni di Dio e Ruggi D'Aragona University Hospital, 84126 Salerno, Italy

**Keywords:** Nanotechnology, Silver nanoparticles (AgNPs), Antiviral activity, HSV-1, SARS-CoV-2, Green synthesis, Biomedical applications

## Abstract

The growing threat of viral infections requires innovative therapeutic approaches to safeguard human health. Nanomaterials emerge as a promising solution to overcome the limitations associated with conventional therapies. The eco-friendly synthesis of silver nanoparticles (AgNPs) currently represents a method that guarantees antimicrobial efficacy, safety, and cost-effectiveness. This study explores the use of AgNPs derived from the peel (Lp-AgNPs) and juice (Lj-AgNPs) *Citrus limon “Ovale di Sorrento”*, cultivars of the Campania region. The antiviral potential was tested against viruses belonging to the *Coronaviridae* and *Herpesviridae*. AgNPs were synthesized by reduction method using silver nitrate solution mixed with aqueous extract of *C. limon* peel and juice. The formation of Lp-AgNPs and Lj-AgNPs was assessed using a UV–Vis spectrophotometer. The size, ζ-potential, concentration, and morphology of AgNPs were evaluated by dynamic light scattering (DLS), nanoparticle tracking analysis (NTA), and field emission-scanning electron microscopy (FE-SEM). Cytotoxicity was evaluated in a concentration range between 500 and 7.8 µg/mL on VERO-76 and HaCaT cells, with the 3-(4,5-dimethylthiazol-2-yl)-2,5-diphenyltetrazolium test bromide (MTT). Antiviral activity consisted of virus pre-treatment, co-treatment, cellular pre-treatment, and post-infection tests versus HSV-1 and SARS-CoV-2 at a multiplicity of infections (MOI) of 0.01. Plaque reduction assays and real-time PCR provided data on the antiviral potential of tested compounds. Lp-AgNPs and Lj-AgNPs exhibited spherical morphology with respective diameters of 60 and 92 nm with concentrations of 4.22 and 4.84 × 10^10^ particles/mL, respectively. The MTT data demonstrated minimal cytotoxicity, with 50 % cytotoxic concentrations (CC_50_) of Lp-AgNPs and Lj-AgNPs against VERO cells of 754.6 and 486.7 µg/mL. Similarly, CC_50_ values against HaCaT were 457.3 µg/mL for Lp-AgNPs and 339.6 µg/mL for Lj-AgNPs, respectively. In the virus pre-treatment assay, 90 % inhibitory concentrations of HSV-1 and SARS-CoV-2 were 8.54–135.04 µg/mL for Lp-AgNPs and 6.13–186.77 µg/mL for Lj-AgNPs, respectively. The molecular investigation confirmed the antiviral data, recording a reduction in the UL54 and UL27 genes for HSV-1 and in the Spike (S) gene for SARS-CoV-2, following AgNP exposure. The results of this study suggest that Lp-AgNPs and Lj-AgNPs derived from *C. Limon* could offer a valid ecological, natural, local and safe strategy against viral infections.

## Highlights

• Nanotechnology offers new ways to prevent and diagnose diseases using advanced methods.
• Green synthesis of plant-based AgNPs is safer and more environmentally friendly, reducing cell toxicity.
• Silver nanoparticles fight bacteria, viruses and fungi, providing solutions for infection control.
• Our study demonstrated the AgNP synthesis from *C. limoni*, and their virucidal effects.

## Graphical abstract

**Figure:**
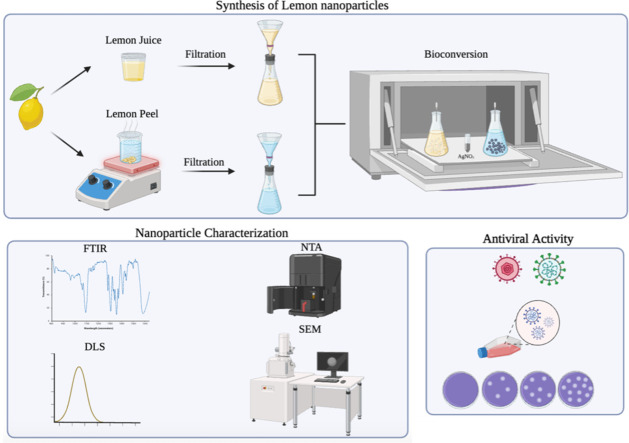

## 1 Introduction

Nanotechnology represents an innovative path for advancing preventive and diagnostic methodologies in treating diseases (Rezaei et al., 2019; Lamparelli et al., 2023). Nanoparticles (NPs) are nanoscale particles ranging from 1 to 100 nm (Gupta and Xie, 2018). Due to their physical (plasmon resonance, fluorescence enhancement, electrical conductivity) and chemical (catalytic activity enhancement and high reactivity) properties, silver metallic nanoparticles (AgNPs) are arousing considerable interest in the field of medical sciences (Isa et al., 2023; Cao et al., 2011; Zhang et al., 2016). AgNPs particularly stand out in this area due to their multiple biological abilities. They exhibit robust anti-inflammatory, anti-tumor and antimicrobial properties (Gorabi et al., 2019; Ion et al., 2021).

Numerous evidence highlights the effectiveness of AgNPs against bacteria, viruses and fungi. Although silver has long been recognized for its antibacterial properties, the effectiveness of AgNPs is significantly increased due to the increased surface area-to-volume ratio compared to bulk silver, facilitating greater interaction with bacterial cell membranes and intracellular components. This characteristic facilitates a more controlled and efficient release of silver ions (Ag^+^), which are widely recognized as the principal agents underlying silver's antimicrobial properties, thereby reducing the likelihood of adverse toxic effects (Yin et al., 2020). The antimicrobial activity of AgNPs against Gram-positive bacteria (*Staphylococcus epidermidis, Staphylococcus aureus, Enterococcus faecalis,* etc.), Gram-negative bacteria (*Escherichia coli, Klebsiella pneumoniae, Salmonella enterica Typhimurium*) and fungi (*Candida albicans*, C*. parapsilosis*, etc.) is known (Gouyau et al., 2021; Dias de Emery et al., 2023; Arsène et al., 2023; Mare et al., 2021). Although the precise mechanism underlying the antibacterial effects of AgNPs remains incompletely understood, several hypotheses have been proposed to rationalize their antibacterial activity. They possess the ability to continuously release silver ions, which are implicated in microbial eradication. These ions show an affinity for sulfur and proteins, thus facilitating their adhesion to the cytoplasmic membrane. Once attached, these ions increase the permeability of the cytoplasmic membrane, ultimately culminating in the rupture of the bacterial envelope. After the internalization of free silver ions by the cells, several subsequent events occur: i) denaturation of the ribosomes, thus preventing protein synthesis; ii) interference with the production of ATP, due to the inactivation of respiratory enzymes on the cytoplasmic membrane; iii) stimulation of the production of reactive oxygen species, resulting in membrane rupture and DNA modification; iv) interruption of DNA replication: both silver ions and reactive oxygen species bind to DNA, preventing its replication and cell proliferation (Anees Ahmad et al., 2020). In addition to the properties mentioned above, AgNPs act on multiple targets, overcoming the problem relating to the development of microbial resistance (Bogart and Licht, 1986; Burdușel et al., 2018). Studies relating to the antiviral activity of AgNPs are more limited. AgNPs have demonstrated efficacy against viruses that possess both DNA (e.g., *Herpesviridae*) and RNA (e.g., *Coronaviridae*) genomes, often through direct interaction with viral structure. This interaction can lead to damage to the viral capsid/envelope or interfere with viral surface proteins thus hindering the viral entry phase (Ratan et al., 2021; Luceri et al., 2023). Srisrimal and colleagues recently highlighted the antiviral efficacy of AgNPs against *Herpes simplex virus type 1* (HSV-1) and *Influenza A virus subtype H1N1*, recording a concentration inhibiting 50 % of the infection at 19.6 μg/mL and over 90 % at 17 μg/mL, respectively (19). The synthesis of silver nanoparticles (AgNPs) encompasses various methodologies, including chemical, physical, and biological approaches.

The main disadvantage associated with the AgNPs chemical and physical synthesis lies in their considerable cellular toxicity, which limits the therapeutic window and precludes their application in pharmacology (Ferdous and Nemmar, 2020). To overcome this hindrance, chemical/physical synthesis methods have been supplanted by a "green" synthesis approach, using plant sources (leaves, roots, stems, flowers, etc.) to produce AgNPs. The use of environmentally friendly materials and cost-effectiveness constitute the most important pillars behind the green synthesis approach (Nie et al., 2023). Indeed, plant-derived NPs are safe, environmentally friendly, biocompatible, economical, rapidly synthesized, and recognized as antioxidant, anti-inflammatory, and stabilizing agents (Simon et al., 2022). Considering this, the green method appears to be a more efficient alternative to chemical and physical approaches (Hano and Abbasi, 2021).

Contextually, our study involved the green synthesis of AgNPs using *Citrus limon “Ovale di Sorrento”*, (*C. limon*), a cultivar belonging to the *Rutaceae* family. The latter is cultivated with agricultural practices that promote environmental sustainability, avoiding the use of synthetic pesticides, chemical fertilizers and other harmful substances. The NPs were synthesized from two sources, lemon peel and lemon juice, through the bioconversion of silver nitrate salts. Assessment of the virucidal effect against HSV-1 and Severe Acute Respiratory Syndrome Coronavirus-2 (SARS-CoV-2) revealed a high-performance activity of the green synthesis, demonstrating the benefits associated with the use of photoproducts as potential sources for the AgNPs production in the biomedical domain.

## 2 Material and methods

### 2.1 Citrus limon extracts and NP green synthesis

The bioactive molecules derived from *C. limon* peel and juice were obtained following the extraction procedure of Nisha et al. and Mahiuddin et al., respectively (Nishihara et al., 1987; Mahiuddin and Ochiai, 2021). The sample was collected from a local farmer in Sorrento (Italy) and washed thoroughly with deionized water. The lemon peel was cut into small pieces, 8 g of material was weighed and transferred to 80 mL of distilled water, heated at 80 °C for 10 min, and cooled at room temperature for 30 min. The extract obtained was filtered through Whatman filter paper n.1 and further filtration 0.45 μm. The filtrate was stored at 4 °C until use. The juice was collected by squeezing the lemon followed by centrifugation and filtration with Whatman n.1 filter paper and 0.45 μm filter. The extract, stored at 4 °C until use, was adjusted to pH 7.5 using 7 M aqueous NaOH before the bioconversion reaction. For NP green synthesis, 3 mL of peel extract and 3 mL of juice were added to 30 and 60 mL of 1 mM AgNO_3_ aqueous solution, in a 250 mL flask under constant vigorous stirring, at room temperature, in the dark environment, for about 2 h. After 40 min, a color change of the suspension from colorless to dark brown/golden brown confirmed the biosynthesis of the AgNPs. The reaction was monitored for the next 4 days to exclude the presence of precipitates. Then the solution was centrifuged, washed with H_2_O to remove excess AgNO_3_, and the pellet was dried at 40 °C to obtain powdered AgNPs. The reaction was monitored with UV–Vis spectroscopy in the wavelength from 300 to 600 nm to observe the reduction of pure Ag^+^ ions. The obtained lemon peel (Lp-AgNPs) and lemon juice (Lj-AgNPs) NPs were resuspended in sterile distilled water (2.5 mg/mL) and sonicated before use.

### 2.2 Dynamic light scattering (DLS) and nanoparticle tracking analysis (NTA)

The Lp-AgNPs and Lj-AgNPs Z-average size (Z-ave) and polydispersity index (PDI) were defined by DLS analysis, using a Zetasizer Nano S instrument (Malvern PANalytical, Worcestershire, UK). The Z-ave value defines the average diameter of the NPs while PDI provides information on the particle size distribution. A volume of 1 mL for each sample was transferred in cuvettes and gently mixed to provide a homogeneous solution. Data were analyzed using Dispersion Technology Software (DTS) (V7.01) provided by Malvern Zetasizer Nano-ZS +. Measurements obtained via DLS were augmented using a NanoSight NS300 instrument (Malvern Panalytical, Worcestershire, UK), which allowed NP concentration to be defined. Samples diluted 1:10.000 were infused into the NanoSight instrument using a syringe pump with a rate of “40″ and a camera level of “10″. A total of 5 readings lasting 60 s each were acquired. Frames were analyzed with NTA software (Malvern Instruments, version 3.2, Worcestershire, UK) which defines the average size, mode, and concentration (particles/mL).

### 2.3 Zeta potential and fourier transform infrared spectra

To assess the stability of AgNPs' solutions, the ζ-potential was determined using the electrophoretic light scattering method (ELS) through a Zetasizer Nano S instrument (Malvern PANalytical, Worcestershire, UK). The ζ-potential measurements were carried out at room temperature employing disposable cuvettes (DTS1070, Malvern PANalytical) filled with 1 mL of the sample. To ensure accuracy, all measurements were repeated three times, with a 60-second equilibration period between each measurement. The software was configured to the automatic acquisition mode. Fourier transform infrared (FTIR) spectra were recorded employing a Bruker Vertex 70 spectrometer (Bruker Corporation, Germany) equipped with a deuterated triglycine sulfate detector (DTGS) and a Ge/KBr beam splitter. The spectra were collected over a range from 4000 to 400 cm−1 through 128 scans at a resolution of 2 cm−1. The solid samples were mixed with KBr and compressed into pellet form. Analysis of the spectra was performed using OPUS 6.0 Software for Windows.

### 2.4 Field emission scanning electron microscopy (FE-SEM)

The surface and morphology of silver nanoparticles were observed using field emission-scanning electron microscopy (FE-SEM, model LEO 1525, Carl Zeiss SMT AG, Oberkochen, Germany). Sample droplets were placed onto a double-sided adhesive carbon tape that was previously attached to an aluminum stub, and subsequently, they were subjected to vacuum drying. Finally, a thin layer of gold film (250 Å) was applied to the dried droplets using a sputter coater (model 108 A, Agar Scientific, Stansted, United Kingdom) (Lamparelli et al., 2022). The following standard settings were employed for the FESEM imaging: acceleration Voltage of 15 kV; working Distance (WD) of 8 mm; detector Secondary Electron (SE) imaging mode; magnification starting at 500x; aperture size adjusted for optimal resolution (but 30 µm, most used). Regarding sample Preparation, the silver nanoparticles were drop-casted onto a conductive substrate and allowed to dry before imaging to minimize charging effects; the beam current has been optimized for sensitivity and resolution without damaging the sample; the scanning speed has been adjusted for the desired image quality; focus and stigmation has been optimized for sharp and well-focused images. The acquired FESEM images were analyzed using Image J processing software to enhance contrast and sharpness for better visualization and analysis of the silver nanoparticles.

### 2.5 Cell viability assessment

To define the Lp-AgNPs and Lj-AgNPs cytotoxic value, the 3-(4,5-dimethylthiazol-2-yl)−2,5-diphenyltetrazolium bromide (MTT) assay was performed on Human Epidermal Keratinocytes (HaCaT) and Kidney Epithelial cells (VERO-76) extracted from African green monkey (*Cercopithecus aethiops*). Cells were purchased from American Type Culture Collection (ATCC, Manassas, VA, USA) and cultured in Dulbecco's Modified Eagle Medium (Thermo Fisher Scientific, Waltham, MA, USA) with 4.5 g/L of glucose, 2 mM of l-glutamine, 100 IU/mL of penicillin-streptomycin solution, 10 % fetal bovine serum (FBS; Thermo Fisher Scientific, Waltham, MA, USA) in a humidified atmosphere with 5 % CO2 at 37 °C. A density of 2 × 10^4^ cells/well was seeded in a 96-well plate and the following day exposed to Lp-AgNPs and Lj-AgNPs, in the range of 500–15.6 μg/mL. After 24 h, the compound was removed and the MTT solution (Sigma-Aldrich, St. Louis, MO, USA) was added for 3 h at 37 °C. Then, the formed formazan crystals were solubilized with 100 % DMSO, and the absorbance related to cell viability was calculated at Optical density (OD) 570 nm using a microplate reader (Tecan, Männedorf, Switzerland). Unexposed cells represented the negative control (CTRL−), while cells treated with DMSO (100 %) constituted the positive control (CTRL+).

### 2.6 Antiviral activity

To inhibit viral infectivity and understand the NP action mode, four Plaque Reduction Assays were performed against two different models: HSV-1 and SARS-CoV-2. Viruses were propagated in VERO-76 cells and stored at −80 °C until use. The assays performed as previously described (Dell'Annunziata et al., 2022; Dell'Annunziata et al., 2023), included: i) Co-treatment, where viruses and NPs were simultaneously added to the cell monolayer; ii) Virus pre-treatment, which involved the viral strain exposure to Lp-AgNPs and Lj-AgNPs following cellular infection; iii) Post-infection, in which previously infected cells were treated with serial dilutions of NPs; iv) Cell pretreatment whereby cells are treated with serial dilutions of Lp-AgNPs and Lj-AgNPs before viral inoculation. For both strains, infection was conducted at a multiplicity of infection (MOI) of 0.01 plaque forming units (PFU)/cell, with an adsorption time of 1 hour at 37 °C. Then, the cells were washed to remove the extracellular viruses and covered with carboxymethylcellulose (3 %, Sigma-Aldrich, St. Louis, MO, USA) added to DMEM, for a replication time of 24/48 h. Then, the cell monolayer was fixed with 4 % formaldehyde (Sigma-Aldrich, St. Louis, MO, USA) and stained with 0.5 % crystal violet solution (Sigma-Aldrich, St. Louis, MO, USA). Each viral lysis zone constituted a plaque-forming unit, counted to define viral inhibition by comparing treated versus untreated wells, according to the following formula:

$$\;\%\;\mathrm{Viral}\;\mathrm{inhi}\mathrm{biti}\mathrm{on}\;=\;100-\left[100\times \left(\frac{\mathrm{plaq}\mathrm{ues}\;\mathrm{coun}\mathrm{ted}\;\mathrm{in}\;\mathrm{the}\;\mathrm{test}\;\mathrm{samp}\mathrm{le}}{\mathrm{plaq}\mathrm{ues}\;\mathrm{coun}\mathrm{ted}\;\mathrm{in}\;\mathrm{the}\;\mathrm{CTRL}-}\right)\right]$$

For HSV-1, the positive control (CTRL+) was melittin at 5 µM during co-treatment and pre-treatment virus; dextran sulfate at 1 µM before exposing the virus to cells and acyclovir at 5 µM after exposing the cells to the virus. For SARS-CoV-2, *Ficus rubiginosa* leaf extracts at 10 μg/mL were used during co-treatment and before exposing the virus to cells, ivermectin at 10 µM before exposing the cells to the virus and remdesivir at 10 µM after exposing the cells to the virus.

### 2.7 Evaluation of viral gene expression

To confirm the results obtained through the plaque reduction assays, the molecular analysis was conducted in virus pretreatment. Genomic RNA was extracted by TRIzol (Thermo Fisher Scientific, Waltham, MA, USA) and quantified by NanoDrop (NanoDrop 2000, Thermo-Fisher Scientific). One microgram of RNA was reverse transcribed into cDNA using 5 × All-In-One RT Master Mix (Applied Biological Materials, Richmond, VA, Canada) and amplified by Real-time PCR (LightCycler® system Roche, Basel, Switzerland). For HSV-1, the expression of specific viral genes, UL54 (immediate early gene, F: 5′-TGGCGGACATTAAGGACATTG-3′, R: 3′-TGGCCGTCAACTCGCAG-5′) and UL27 (late gene, F: 5′-GCCTTCTTCGCCTTTCGC-3′, R: 3′-CGCTCGTGCCCTTCTTCTT-5′), for SARS-CoV-2 the expression of the gene S (spike protein, F: 5′-AGGTTGATCACAGGCAGACT-3′, R: 3′-GCTGACTGAGGGAAGGAC-5′) were evaluated. Relative target threshold cycle (Ct) values were normalized to the housekeeping gene glyceraldehyde 3-phosphate dehydrogenase (GAPDH, F: 5′-CCTTTCATTGAGCTCCAT-3′, R: 3′-CGTACATGGGAGCGTC-5′) and calculated using the 2^-ΔΔCt^ method.

### 2.8 Statistical analysis

All tests were performed in biological duplicate and technical triplicate. Data are expressed as mean ± standard deviation (SD) calculated by GraphPad Prism ver. 9 for Windows (Software GraphPad, San Diego, CA, USA, www.graphpad.com, accessed on 1 December 2023). The one-way ANOVA statistic was followed by Dunnett's multiple comparisons test and a *p-value* ≤ 0.05 was considered significant. The −50 and −90 % cytotoxic concentrations (CC_50_, CC_90_) and the −50 and −90 % inhibitory concentrations (IC_50_ and IC_90_) were calculated by nonlinear regression analysis using GraphPad Prism software. These values were necessary to calculate the therapeutic index (TI), which indicates the safety between the dose inhibiting viral infectivity and the lethal dose. Specifically, the TI was the ratio of cytotoxicity and antiviral efficacy (CC_50_/IC _50_). High TI values indicate greater safety of the compound.

## 3 Results

### 3.1 Synthesis and characterization of C. limon-NPs

**Fig. 1:**
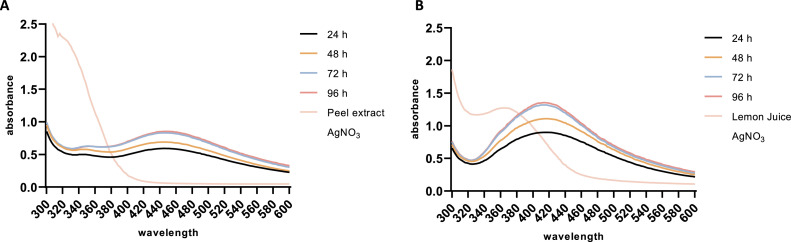
UV–vis absorbance spectra monitoring the biosynthesis and stability of Lp-AgNPs (A) and Lj-AgNPs (B) for 4 days.

The spectral properties of biosynthesized NP spectra were investigated by measuring the absorbance within the wavelength range of 300–600 nm. Following bioconversion, the characteristic surface plasmon resonance (SPR) of AgNPs was observed at 440 nm for Lp-AgNPs and 420 nm for Lj-AgNPs (Fig. 1). Notably, the aqueous solution containing 1 mM AgNO_3_ did not record SPR throughout the experimental period, while lemon peel extract and juice showed absorbance peaks at 340 and 380 nm, respectively. Over a monitoring period of 4 days, both Lp-AgNPs and Lj-AgNPs presented without precipitates and maintained consistent absorbed wavelength.

**Fig. 2:**
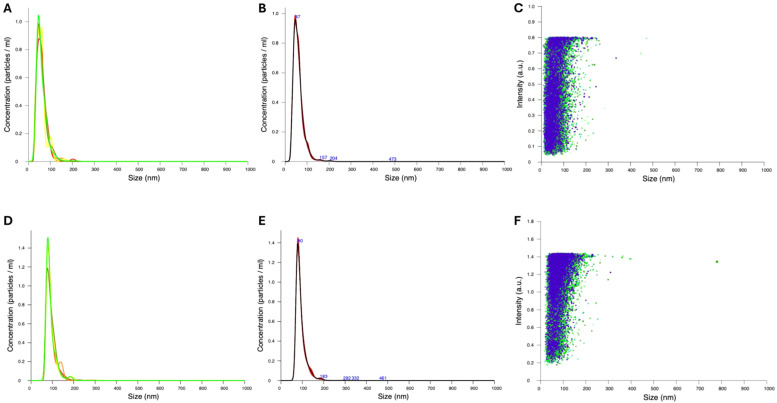
NTA analysis of NPs. Concentration/size graph FTLA (Finite Track Length Analysis) of Lp-AgNPs (A-B) and Lj-AgNPs (D-E), Intensity/size graph of Lp-AgNPs (C) and Lj-AgNPs (F).

The physical characterization parameters provided insights into the suitability of the NPs for assessing biological activity. NP size and ζ-potential were determined through DLS (Supplementary Figure S1) and NTA (Fig. 2). Consistent results from both analyses indicated an average particle diameter of 60.0 ± 25.3 nm and 92.0 ± 24.9 nm with a modal value of 46.1 nm and 79.3 nm for Lp-AgNPs and Lj-AgNPs, respectively. The NP concentration was 4.22 ± 2.71 × 10^10^ and 4.84 ± 2.22 × 10^10^ particles/mL, respectively. Additional chemical-physical characterization was performed by analyzing the ζ-potential of Lp-AgNPs and Lj-AgNPs. The results revealed that both Lp-AgNPs and Lj-AgNPs exhibited a negative ζ-potential, with values ranging from −41.2 (± 7.76) to −50.4 (± 7.52) mV, respectively (Figure S1 b,d).

**Fig. 3:**
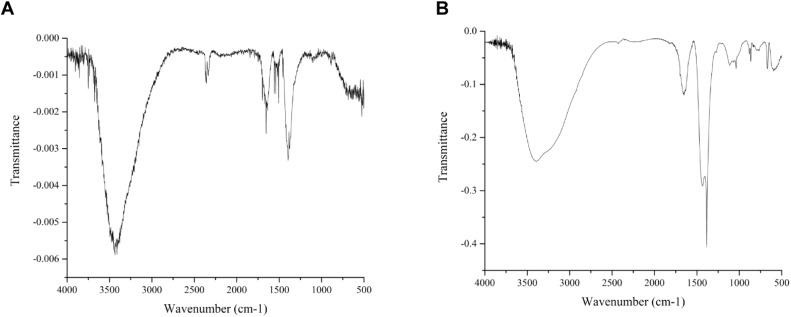
FTIR spectra of silver nanoparticles (AgNPs) derived from the peel (Lp-AgNPs) (A) and juice (Lj-AgNPs) (B) *C. limon*. The FTIR spectra were recorded ranging from 4000 to 400 cm−1 (wavenumber).

FT-IR spectroscopy was employed to investigate the chemical bonds and detect the presence of functional groups in the silver nanoparticles. When applied to silver nanoparticles, FT-IR spectroscopy can provide valuable information about surface chemistry, including the nature of stabilizing agents, surface coatings, and any functional groups present. The FTIR spectra illustrated in Fig. 3 for silver nanoparticles (AgNPs) synthesized from the peel (Fig. 3A) and juice (Fig. 3B) of *C. limon* offer a detailed view of the surface chemistry and functional groups present on these nanomaterials. In detail, O—H Stretching Vibration (3400–3200 cm⁻¹) is typically attributed to the hydroxyl groups present in non-dissociatively adsorbed water molecules on the nanoparticle surface. Such water molecules can either be residual from the synthesis process or adsorbed from the environment. The presence of these hydroxyl groups indicates a hydrophilic nature, which can influence the dispersion and stability of the nanoparticles in aqueous environments. A notable band around 1630 cm⁻¹ corresponds to the bending (scissoring) vibration of water molecules. This observation further confirms the presence of adsorbed water on the nanoparticle surface (Radzikowska-Büchner et al., 2023). The identification of this band, alongside the O—H stretching vibration, underscores the significant role of water in the surface chemistry of the AgNPs, potentially impacting their interaction with other substances and overall stability. C—N and C—O Stretching Vibrations (1380 cm⁻¹ and 1100 cm⁻¹) are indicative of C—N and C—O stretching vibrations, respectively. These functional groups are commonly associated with organic molecules, such as amino acids, proteins, and other phytochemicals, likely present in the citrus peel and juice extracts. These compounds can act as capping agents, stabilizing the nanoparticles by binding to the silver surface and preventing aggregation. The C—N stretch suggests the presence of nitrogen-containing compounds, possibly proteins or other biomolecules, while the C—O stretch is indicative of oxygenated functional groups such as alcohols, ethers, or carboxylic acids. The presence of peaks in the 500–600 cm⁻¹ region is associated with Ag-O bond vibrations. This indicates an interaction between silver atoms and oxygen-containing species on the nanoparticle surface. These Ag-O bonds suggest the possibility of an oxide layer or the presence of oxidized silver species, which could arise during the synthesis process or upon exposure to the atmosphere. This interaction is crucial as it can affect the chemical reactivity, catalytic properties, and potential antimicrobial activity of the AgNPs. The formation of a silver oxide layer can also influence the optical properties of the nanoparticles, which are important in applications like sensing and imaging. The organic molecules derived from Citrus limon extracts not only stabilize the nanoparticles but also confer additional functionalities that can be exploited in various applications. For instance, the presence of biomolecules may enhance biocompatibility, making these nanoparticles suitable for biomedical applications, including drug delivery and antimicrobial treatments. Furthermore, the Ag-O interactions suggest potential uses in catalysis and environmental remediation, where oxidative properties are beneficial.

**Fig. 4:**
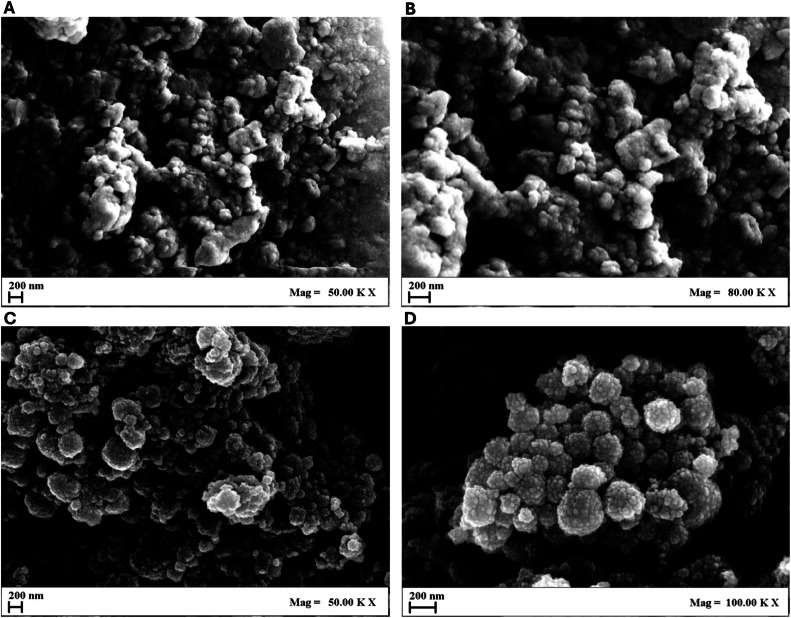
FE-SEM micrographs of Lp-AgNPs at 50.000 × (A) and 80.000 × (B) magnification and Lj-AgNPs at 50.000 × (C) and 100.000 × (D) magnification.

Furthermore, the morphology of both Lp-AgNPs and Lj-AgNPs was verified through FE-SEM analysis (Fig. 4). Small particles were identified with a size distribution in line with the values recorded in NTA and DLS. The Fe-SEM micrographs depicted the NP with a spherical morphology and wrinkled surfaces, assembled to form nanoclusters.

### 3.2 Cell viability alteration

**Fig. 5:**
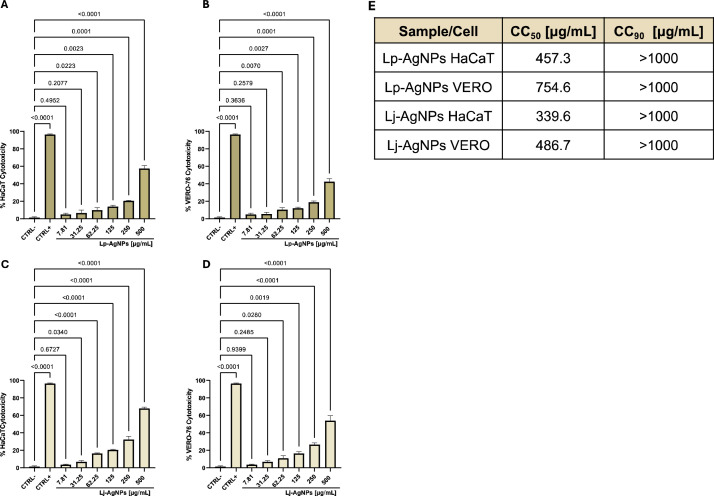
MTT assay exposing Lp-AgNPs to HaCaT (A) and VERO-76 (B) cells and Lj-AgNPs to HaCaT (C) and VERO-76 (D) cells. Table 1(E) CC_50_ and CC_90_ of Lp-AgNPs and Lj-AgNPs exposed to HaCaT and VERO-76 cells. The mortality rate was evaluated compared to unexposed cells, which represent the CTRL-. The CTRL+ was represented by 100 % DMSO and induced 100 % mortality. Results represent the mean ± SD of three independent experiments. Statistical differences were analyzed through one-way ANOVA and Dunnett's multiple comparisons test. The *p-value* ≤ 0.05 was considered significant. Table 1E. CC_50_ and CC_90_ values of Lp-AgNPs and Lj-AgNPs exposed to HaCaT and VERO-76 cells.

To evaluate the toxicity of Lp-AgNPs and Lj-AgNPs, HaCaT cells were selected as the human cell line and VERO-76 cells were needed for subsequent antiviral tests. The cells were exposed to the NPs in concentrations ranging from 500 to 7.81 µg/mL for 24 h (Fig. 5). Subsequently, the cytotoxic effect was evaluated in comparison to unexposed cell control (CTRL-). The findings indicated that no tested compound induced complete cell viability alteration within the examined concentration range. In detail, Lp-AgNPs exhibited toxicity of approximately 57.5 and 42.5 % at 500 µg/mL in HaCaT and VERO-76 cells, respectively. Conversely, exposure to the maximum concentration of Lj-AgNPs resulted in cell death rates of 68 and 54 %, respectively. Through nonlinear regression analysis using GraphPad Prism software, the recorded CC_50_ values were 457.3 and 754.6 µg/mL for Lp-AgNPs and 339.6 and 486.7 for µg/mL for Lj-AgNPs, relative to HaCaT and VERO-76 Cells, respectively. Toxicity values below 50 % were selected for assessing antiviral efficacy (Table 1E).

**Table 1:** CC_50_ and CC_90_ values of Lp-AgNPs and Lj-AgNPs exposed to HaCaT and VERO-76 cells.

Sample/Cell	CC_50_ [µg/mL]	CC90 [µg/mL]
Lp-AgNPs HaCaT	457.3	> 1000
Lp-AgNPs VERO	754.6	> 1000
Lj-AgNPs HaCaT	339.6	> 1000
Lj-AgNPs VERO	486.7	> 1000

### 3.3 Antiviral activity

**Fig. 6:**
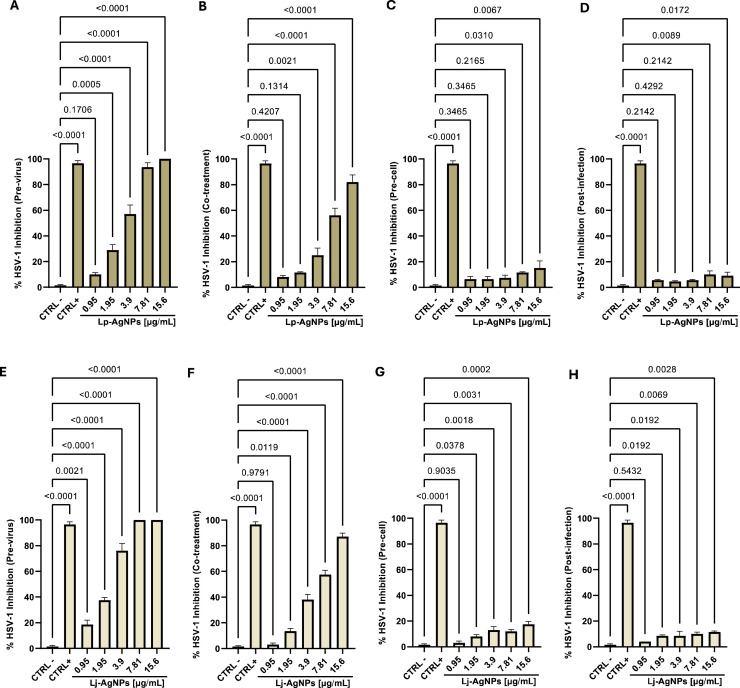
Antiviral effect (expressed as % inhibition) of Lp-AgNPs and Lj-AgNPs (μg/mL) against HSV-1 in different plaque reduction assays: (A-E) virus pretreatment; (B-F) co-treatment; (C-G) Cell pretreatment; (D-H) post-infection. Results represent the mean ± SD of three independent experiments. Statistical differences were analyzed through one-way ANOVA and Dunnett's multiple comparisons test. The *p-value* ≤ 0.05 was considered significant.

The NP virucidal efficacy was investigated via plaque reduction assay and qPCR against HSV-1 (enveloped DNA virus) and SARS-CoV-2 (enveloped RNA virus). In the initial screening against HSV-1, viral particles and compounds were co-incubated with the cell monolayer for 1 hour, resulting in maximum inhibition at a concentration of 30 µg/mL (Data not shown). However, inhibition exceeding 50 % was observed for both compounds at concentrations up to the concentration of 7.81 µg/mL. Nonlinear regression analysis yielded IC_50_ and IC_90_ values of 6.8–24.21 and 5.78–21.72 µg/mL for Lp-AgNPs and Lj-AgNPs, respectively (Table 2). In the virus pretreatment assay, Lp-AgNPs and Lj-AgNPs recorded total inhibition up to 7.8 µg/mL, indicating their virucidal action was clear. IC_50_ and IC_90_ values were 3.09–8.54 and 2.29–6.13 µg/mL for Lp-AgNPs and Lj-AgNPs, respectively. The assays suggested a potential impact of the NPs in the extracellular infection phases directly targeting viral particles. However, the possibility of action on host cells and/or intracellular receptors was not excluded. Therefore, cell pretreatment and post-infection assay were conducted, revealing no inhibition within the sub-toxic concentration range (Fig. 6).

**Table 2:** IC_5_**_0_**, IC_90_ and TI of Lp-AgNPs and Lj-AgNPs against HSV-1 in virus pretreatment, cell pretreatment, co-treatment, and post-treatment assays.

Sample-Test	IC_50_ µg/mL	IC_90_ µg/mL	TI
Lp-AgNPs Co-treatment	6.8	24.21	110.9
Lp-AgNPs Pre-virus	3.09	8.54	224.2
Lp-AgNPs Pre-cell	>250	>250	/
Lp-AgNPs Post-infection	>250	>250	/
Lj-AgNPs Co-treatment	5.78	21.72	84.20
Lj-AgNPs Pre-virus	2.29	6.13	212.53
Lj-AgNPs Pre-cell	>250	>250	/
Lj-AgNPs Post-infection	>250	>250	/

**Fig. 7:**
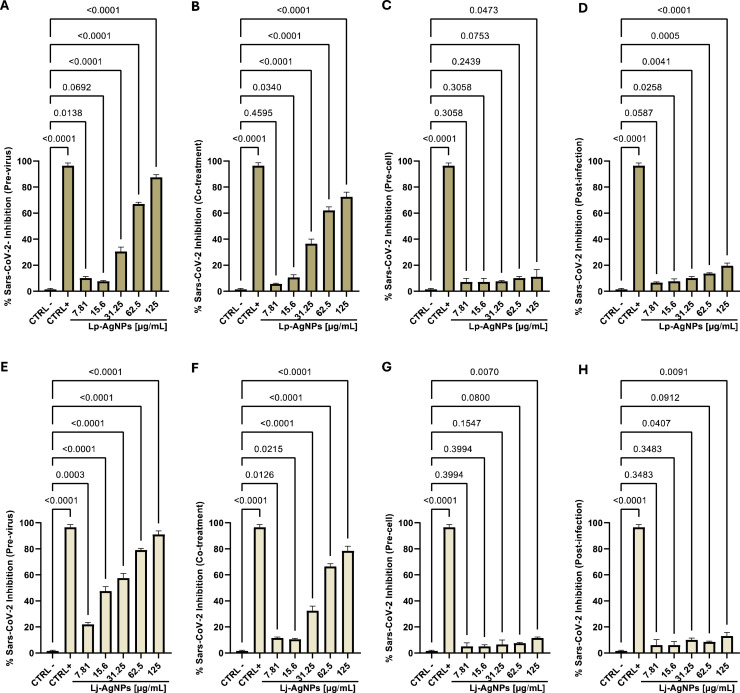
Antiviral activity (expressed as % inhibition) of Lp-AgNPs and Lj-AgNPs (μg/mL) against SARS-CoV-2 in different plaque reduction assays: (A-E) virus pretreatment; (B-F) co-treatment; (C-G) Cell pretreatment; (D-H) post-infection. Results represent the mean ± SD of three independent experiments. Statistical differences were analyzed through one-way ANOVA and Dunnett's multiple comparisons test. The *p-value* ≤ 0.05 was considered significant.

Afterward, efforts have focused on the NPs antiviral evaluation against SARS-CoV-2, chosen as a viral model with RNA genome. The findings revealed a significative inhibition of Coronavirus replication, although with a different efficiency compared to HSV-1. In the co-treatment screening, Lp-AgNPs and Lj-AgNPs demonstrated inhibitory effects of 72.5 and 71.5 % at 125 µg/mL. The detected IC_50_ and IC_90_ values related to Lp-AgNPs and Lj-AgNPs were 50.58–237.67 and 46.90–186.87 µg/mL, respectively. Consistent with the results obtained against HSV-1, the virucidal effectiveness increased up to 87.5 and 91 % in virus pre-treatment. Conversely, no activity was detected in cellular pre-treatment and post-infection (Fig. 7). In detail, the IC_50_ and IC_90_ values related to Lp-AgNPs and Lj-AgNPs were 40.75–135.04 and 27.47–133.87 µg/mL in pre-treatment (Table 3).

**Table 3:** IC_50_, IC_90_ and TI of Lp-AgNPs and Lj-AgNPs against SARS-CoV-2 in virus pretreatment, cell pretreatment, co-treatment, and post-treatment assays.

Sample-Test	IC_50_ µg/mL	IC_90_ µg/mL	TI
Lp-AgNPs Co-treatment	50.58	237.67	14.91
Lp-AgNPs Pre-virus	40.75	135.04	18.52
Lp-AgNPs Pre-cell	>250	>250	/
Lp-AgNPs Post-infection	>250	>250	/
Lj-AgNPs Co-treatment	46.90	186.77	10.37
Lj-AgNPs Pre-virus	27.47	133.87	17.71
Lj-AgNPs Pre-cell	>250	>250	/
Lj-AgNPs Post-infection	>250	>250	/

**Fig. 8:**
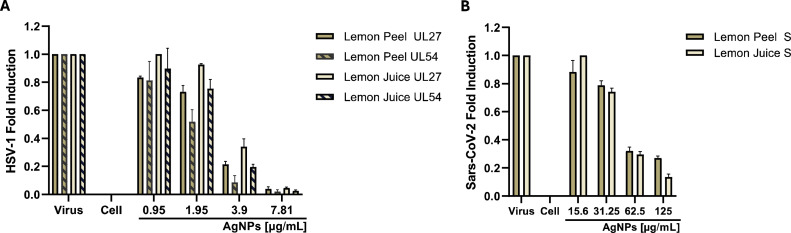
Analysis of HSV-1 [UL27 and UL54] (A) and SARS-CoV-2 [S] (B) expression gene levels after treatment with Lp-AgNPs and Lj-AgNPs. Data represent the mean ± standard deviation (SD) of three independent experiments.

The plaque reduction assay provided the information necessary to define the effective dose and explore the specific phase of viral infection targeted by the compound under analysis. Nevertheless, it is imperative to confirm these findings through molecular investigation of the viral gene inhibition. Given the demonstrated efficacy of Lp-AgNPs and Lj-AgNPs during extracellular infection phases, molecular assays were conducted to evaluate the expression of the UL27 and UL57 gene for HSV-1 and of the S gene for SARS-CoV-2 (Fig. 8). Concerning HSV-1, Lp-AgNPs and Lj-AgNPs elicited complete inhibition at the highest tested concentration. In a dose-dependent manner, an increase in the expression of viral genes was recorded up to 1.95 μg/mL, where gene expression equaled that of the control virus. Conversely, the expression of the gene S at 125 μg/mL was approximately 5 times lower compared to cells infected, with a fold induction of 0.86 at the dose of 31.25 μg/mL. In both conditions the qPCR analysis mirrored the plaque reduction assay, confirming the action of Lp-AgNPs and Lj-AgNPs against enveloped viruses, possessing both DNA and RNA genomes.

The last phase of the study aimed to determine the most suitable compound by considering two important parameters: i) toxic cellular dose and ii) inhibitory viral dose. To accomplish this, the TI was calculated, representing the ratio between the toxic dose and the effective dose. The reference values for defining a safe drug depend on the specific clinical context and the drug's nature. Generally, a therapeutic index exceeding 10 is considered safe, while values below 10 indicate a potential toxic risk. The calculated indices, detailed in Table −2 for HSV-1 and −3 for SARS-CoV-2, highlighted that although Lj-AgNPs showed greater antiviral efficacy, Lp-AgNPs were considered safer. Specifically, in virus pretreatment, the TI values were 224.2–212.53 in HSV-1 infection and 18.52–17.71 in SARS-CoV-2 infection, associated with Lp-AgNPs and Lj-AgNPs, respectively.

## 4 Discussion

The research and development of new drugs represent a crucial frontier in the challenge against viral infections (Gonçalves et al., 2021). The rapid evolution and intricate nature of viral diseases necessitate constant effort in the search for effective and safe therapeutic interventions, emerging as a paramount concern for the scientific community (Cook and Lauer, 2021). Furthermore, available treatments are limited in terms of efficacy and tolerability, contributing to the spread of drug-resistant viral strains which represents a worrying threat (Salam et al., 2023). To date, using NPs synthesized from green sources emerges as a promising and innovative approach to the challenge of viral infections. NPs obtained from natural sources, such as plants, bacteria, or fungi, offer numerous advantages, including biocompatibility, low toxicity, and environmental sustainability (Qasim et al., 2023). They demonstrate documented antimicrobial and antiviral properties and can exert their effects through several mechanisms, including viral replication inhibition, interference with the adhesion of the virus to host cells, and stimulation of the immune system to combat infection (Gurunathan et al., 2020). Additionally, the facile engineering and functionalization capabilities of NPs enable the optimization of their therapeutic applicability (Yetisgin et al., 2020). This study focused on the green synthesis of AgNP, utilizing extracts from two sources of *C. limoni* (peel and juice) through bioconversion with AgNO_3_. The reaction occurred after about 40 min, verified by a solution change from colorless to dark brown/bronze brown for Lp-AgNPs and Lj-AgNPs, respectively. The color change could be associated with reducing Ag^+^ ions during the reaction, after extract addition. The mechanism underlying the synthesis of NPs has not been fully elucidated. However, it is believed that the presence of secondary metabolites generates a redox reaction, thereby triggering the biosynthesis process (Lala, 2021). Furthermore, the same metabolites could be responsible for the stabilization of the NPs (Kulkarni et al., 2023). The synthesis was monitored for four days via UV–visible spectrophotometry, revealing a characteristic bell-shaped curve with absorption at 440 and 420 nm, for Lp-AgNPs and Lj-AgNPs. SPR provided insight into the structure and properties of the NPs, where the presence of a single absorption peak suggested the synthesis of particles with similar characteristics. Otherwise, multiple absorption peaks indicate the presence of particles with diverse shapes in the solution. Subsequent DLS and NTA analysis were employed for particle size analysis, revealing average diameters of 60 nm for Lp-AgNPs and 92 nm for Lj-AgNPs, respectively. The polydispersity index values of 0.387 for Lp-AgNPs and 0.295 for Lj-AgNPs indicated the presence of quite monodisperse particles, suggesting sufficient uniformity in size distribution. Moreover, the presence of the negative ζ-potential for both NPs is important as it promotes electrostatic repulsion among the nanoparticles, thereby enhancing formulation stability and extending shelf-life. Indeed, charged nano-carriers tend to have a reduced propensity for interaction and aggregation over time.

FE-SEM micrographs confirmed the existence of spherical-shaped structures with a tendency to aggregate. The characterization results obtained were by existing experimental evidence documented in the literature, validating the reliability and consistency of the synthesized NPs. A study conducted by Alkhulaifi and colleagues investigated the antibacterial effect of green NPs from lemon peels (Alkhulaifi et al., 2020). The biosynthesis reaction induced a notable color change of the solution to brown, as detected by the UV-visible spectrophotometry, which revealed an absorption peak at 437 nm. DLS analysis showed an aerodynamic diameter of 59.74, with a PDI of 0.463. Similarly, Alaallah et al. reported the synthesis of NPs from lemon juice, yielding a yellowish-brown solution within approximately 30 min of reaction initiation. Characterization of these nanoparticles revealed a spherical shape and SPR absorption measured at 430 nm (Alaallah et al., 2023).

The second experimental step evaluated the antiviral efficacy of the synthesized compounds. First, cell viability analysis was conducted to determine the concentration range that posed no harm to human health. To achieve this, a human cell line (HaCaT) was chosen while a non-human cell line (VERO-76) was used as a model for the high viral tropism. The Lp-AgNPs and Lj-AgNPs toxic effect was dose-dependent with CC_50_ values of 457.3–339.6 and 754.6–486.7 µg/mL in HaCaT and VERO-76, respectively. The observed cytotoxicity could be associated with silver, responsible for several harmful effects on human health, including argyria, kidney damage, allergic reactions, effects on the hematopoietic system, gastrointestinal disorders, etc. (Rezvani et al., 2019; Ahamed et al., 2010; Suthar et al., 2023). It is also demonstrated that exposure to high concentrations or prolonged durations of AgNPs can impact cellular processes through several mechanisms: i) induction of oxidative stress via the generation of reactive oxygen species (ROS) (Yu et al., 2020); ii) interaction with cellular and mitochondrial membranes, leading to organelle destruction and/or the entire cell (Awashra and Młynarz, 2023); iii) promotion of inflammation, through the activation of cytokines and chemokines that stimulate the immune system (Liu et al., 2022); iv) damage to DNA, through direct interaction with nucleic acids or with the enzymes responsible for the replication and

Hence, the antiviral potential was assessed within a concentration range of 125–0.95 µg/mL, using HSV-1 and SARS-CoV-2 as representatives of DNA and RNA genome models, respectively. Phyto sources contain a variety of bioactive molecules with documented antiviral activity and the synthesized Green NPs can increase biological activity and have therapeutic applicability (Habeeb Rahuman et al., 2022). In the present study, the inhibition occurred when the compounds were incubated with the virus before cellular infection. The interpretation of results considered the cytotoxic effect, which was more pronounced in Lj-AgNPs compared to Lp-AgNPs. Therefore, TI was important to understand the association between antiviral effectiveness and safety. TI values of 18.52–17.71 for HSV-1 infection and 224.2–212.53 for SARS-CoV-2 infection were determined for Lp-AgNPs and Lj-AgNPs, respectively. The findings highlighted two key observations: i) Lp-AgNPs were slightly more effective than Lj-AgNPs; ii) both compounds showed a clear difference in antiviral inhibition against the viral strains.

To date, no literature study demonstrated the NP antiviral activity from *C. limon*. Consequently, a direct comparison of our results with existing findings is currently unfeasible. However, it is possible to hypothesize that the mechanism of action involves the viral envelope and/or membrane glycoproteins, given the evident virucidal effect observed during the extracellular phase (Dell'Annunziata et al., 2022). The lipid component destructuring or binding to glycoproteins consequently prevents the viral adsorption phase (Teissier and Pécheur, 2007). The disparity observed in the efficacy of Lj-AgNPs and Lp-AgNPs towards HSV-1 and SARS-CoV-2 could attributed to a dual explanation. Firstly, the viruses differ in size with 200 nm in diameter for HSV-1 and 60–140 nm for SARS-CoV-2 (Rice, 2021; Bullock et al., 2021). Larger viral particles inherently provide a greater contact surface area for interaction with nanoparticles (Rizvi and Saleh, 2018). Moreover, the strains differ in the composition of surface glycoproteins present on the envelope. HSV-1 is characterized by two major surface glycoproteins, namely gB (glycoprotein B) and gD (glycoprotein D) (Jambunathan et al., 2021). Glycoprotein B is involved in the initial adhesion to the host cell and viral fusion (Weed and Nicola, 2017). On the other hand, glycoprotein D is essential for recognizing specific cellular receptors and activating viral entry. Other surface glycoproteins (gH and gL) are involved in viral fusion and membrane invagination (Bivacqua et al., 2023). Otherwise, the predominant surface protein of SARS-CoV-2 is the spike (or S protein), responsible for receptor binding with the angiotensin-converting enzyme 2 (ACE2) (Huang et al., 2020). The spike protein has a trimeric structure with a receptor-binding region (RBD) that specifically binds to ACE2, facilitating viral entry into the host cell (Basavarajappa et al., 2022). Consequently, it is plausible that Lp-AgNPs and Lj-AgNPs exhibit higher binding affinity and specificity to HSV-1 glycoproteins compared to the spike protein. This could result in a reduced dosage requirement for countering infection. Future molecular docking studies will be essential to elucidate the binding mechanisms of the synthesized nanoparticles and delineate the ensuing biological processes.

## 5 Conclusion

The current investigation centered on the NPs green synthesis using *C. limon* extracts (lemon peel and juice) as precursor materials. Our findings, consistent with prior research, highlight the reproducibility and reliability of green synthesis approaches. Characterization techniques demonstrated the formation of small, monodisperse, and spherical-shaped particles. Consequently, the investigation was extended to evaluate the AgNPs antiviral activity against HSV-1 and SARS-CoV-2. The results unveiled a dose-dependent inhibition of viral replication, suggesting potential therapeutic potential against DNA and RNA viruses. Notably, disparities in antiviral efficacy between the two strains were observed. These variations could be attributed to differences in viral size and surface glycoprotein composition, highlighting the complex interplay between NPs and viral targets. In conclusion, our study highlights the promising potential of green-synthesized NPs as a therapeutic route. Harnessing cutting-edge nanotechnologies will enable the development of effective, sustainable, and targeted antiviral therapies, crucial for mitigating the spread of emerging viruses.

## CRediT authorship contribution statement

**Federica Dell'Annunziata:** Conceptualization. **Ekaterine Mosidze:** Data curation. **Veronica Folliero:** Validation. **Erwin P. Lamparelli:** Formal analysis. **Valentina Lopardo:** Investigation. **Pasquale Pagliano:** Methodology. **Giovanna Della Porta:** Resources. **Massimiliano Galdiero:** Project administration. **Aliosha Dzh Bakuridze:** Supervision. **Gianluigi Franci:** Writing – original draft, Funding acquisition.

## Declaration of competing interest

The authors declare that they have no known competing financial interests or personal relationships that could have appeared to influence the work reported in this paper.

## Data Availability

Data will be made available on request.
